# Research rigor and reproducibility in research education: A CTSA institutional survey

**DOI:** 10.1017/cts.2024.10

**Published:** 2024-02-01

**Authors:** Cathrine Axfors, Mario Malički, Steven N. Goodman

**Affiliations:** 1 Stanford University School of Medicine, Stanford Program on Research Rigor & Reproducibility (SPORR), Stanford, CA, USA; 2 Meta-Research Innovation Center at Stanford (METRICS), Stanford University, Stanford, CA, USA; 3 Department of Epidemiology and Population Health, Stanford University School of Medicine, Stanford, CA, USA; 4 Department of Medicine, Stanford University School of Medicine, Stanford, CA, USA

**Keywords:** Rigor, reproducibility, CTSA, NCATS, survey

## Abstract

We assessed the rigor and reproducibility (R&R) activities of institutions funded by the National Center for Advancing Translational Sciences (NCTSA) through a survey and website search (*N* = 61). Of 50 institutional responses, 84% reported incorporating some form of R&R training, 68% reported devoted R&R training, 30% monitored R&R practices, and 10% incentivized them. Website searches revealed 9 (15%) freely available training curricula, and 7 (11%) institutional programs specifically created to enhance R&R. NCATS should formally integrate R&R principles into its translational science models and institutional requirements.

## Introduction

The clinical translatability of laboratory research has long been a concern of the National Institutes of Health (NIH) and was a key motivation for the development of the Clinical and Translational Science Awards (CTSA) program [1]. As Elias Zerhouni stated in 2005, “*The scale and complexity of today’s biomedical research problems demand that scientists move beyond the confines of their individual disciplines and explore new organizational models for team science* [1].” Correspondingly, CTSA hubs are intended to address this problem through education and structures to enhance collaboration of scientists across disciplines and the translational spectrum. The translational pathway model has been expanded and elaborated over the ensuing two decades, under the auspices of the National Center for Advancing Translational Sciences (NCATS), formed in 2011 to administer the CTSA consortium and whose leadership has taken the lead in formalizing and promoting a new “*Science of Translational Science* [2].” This has produced attendant organizational and educational requirements of CTSA-holding institutions, with a goal of increasing the efficiency of the clinical translation.

In 2012, articles by scientists at Bayer and Amgen caught the attention of the scientific community, pointing to poor reproducibility of academic translational research [3,4]. These articles confirmed the concerns of scientists over the preceding decade that the variable quality of the underlying science was a major cause of translational roadblocks, combined with a variety of system features. This provoked a 2014 article by NIH Director Francis Collins, stating that the poor reproducibility of NIH-supported science required “*immediate and substantive action*” and that “*success will come only with full engagement of the entire biomedical enterprise* [5].” This was followed by a series of NIH Rigor and Reproducibility (R & R) requirements for R01 grants (in 2016) [6], T32 grants (in 2020) [7], and data management and sharing plans (in 2023) [8]. Scientific rigor is defined as the strict application of the scientific method to ensure robust and unbiased experimental design, methodology, analysis, interpretation, and reporting of results [6]. A study has good reproducibility if its design, data gathering, analysis, and inferences can be re-run and corroborated. Computational reproducibility refers to the process of obtaining the same (statistical) results by re-running the published analysis using the researchers’ methods and (deposited) code or data [9].

Interestingly, the NIH’s concern with poor research rigor and reproducibility as a contributor to translational failure is not reflected in NCATS translational models or in CTSA hub requirements. There are no requirements specifically related to rigor and reproducibility in the most recent CTSA funding opportunity announcement [10], and minimal language in the 2022 NCATS paper “*Advancing Translational Science Education* [11].” In that paper, the only mention of the R & R comes in a description of a translational scientist as a “*Rigorous researcher*” who “*Conducts research at the highest level of rigor and transparency, possesses strong statistical analysis skills, and designs research projects to maximize reproducibility.*” A new heading of “*Rigor and Reproducibility*” was added to the NCATS Translational Science Principles webpage in April 2023, albeit with minimal details about its operationalization [12].

With the strong NIH emphasis on R & R training and practices as central to the issue of efficient translation, and with the lack of formal R & R institutional requirements from NCATS, we conducted a survey to determine the degree to which CTSA hubs incorporated R & R training and support into their translational research education and support infrastructure.

## Materials and methods

We sent an online survey to principal investigators of all CTSA-funded institutions and searched their websites using “rigor” and “reproducibility” as keywords. The survey had 12 questions related to R & R activities and an open-ended comment section developed by the authors based on their knowledge of the existing activities. Full survey questions, website search strategy, and the list of surveyed institutions are available in the Supplementary File. The survey was sent initially on 6 January 2022, and included three email reminders, as well as two phone call attempts to reach non-respondents. Responses were gathered until August of 2022. The final response rate was 82% (50 of 61 institutions). Survey results are reported as a percentage (and number) of responding institutions (*N* = 50), while resources are collected as a number (and percentage) of all CTSA-funded institutions (*N* = 61). Open-ended answers were inductively classified to identify common themes.

## Results

Survey respondents indicated that 84% (*N* = 42) of institutions had incorporated R & R training into existing programs and courses, 68% (*N* = 34) had training specifically devoted to R & R, 30% (*N* = 15) monitored R & R at their institutions, and 10% (*N* = 5) recognized or incentivized best R & R practices of their researchers (Table 1). In the free text comments section, many respondents indicated that their institutions had “*mandatory research methods*,” “*good laboratory practice,*” or “*responsible conduct of research*” courses, which they considered to fall under R & R even if that terminology was not used in course syllabi. Based on the survey responses and website searches, we identified 33 (54%) institutions with descriptions of R & R training in existing courses, and 34 (56%) with training specifically devoted to R & R. We also identified 34 different R & R resources (e.g., guides, textbooks, courses, etc.) created or externally linked on institutional websites, which included training from nine (15%) institutions with freely available materials. Finally, we identified seven (11%) hubs with programs specifically designed to enhance R & R at their institution (Table 2).

**Table 1.** Rigor and reproducibility (R & R) activities of Clinical and Translational Science Awards hubs reported by survey respondents (*N* = 50)

Activity	*n* (%)
Website with R & R resources	28 (56%)
R & R training incorporated in courses/programs	42 (84%)
R & R devoted training	34 (68%)
Monitoring to assess the implementation of R & R	15 (30%)
Technical or other support for R & R implementation	27 (54%)
Recognition or incentives for best R & R practices	5 (10%)

**Table 2.** Rigor and reproducibility guides, reports or recommendations, programs, and trainings with available course materials identified from Clinical and Translational Science Awards funded institutions

Guides, reports or recommendations	Programs	Trainings
American Statistical Association Recommendations to Funding Agencies for Supporting Reproducible Research	Harvard Medical School HMS R3 Effort - Rigor, Reproducibility and Responsibility at HMS	Rockefeller University CCTS R3 Series - Enhancing Scientific Rigor, Reproducibility, and Reporting
Federation of American Societies for Experimental Biology Enhancing Research Reproducibility	Johns Hopkins University R3 Center for Innovation in Science Education	Indiana Univ-Purdue Univ at Indianapolis Healthcare Triage: Reproducibility in Research, Experimental Design, and Analysis and Reporting, and Reproducibility Crisis podcast
National Academies of Sciences, Engineering, and Medicine Reproducibility and Replicability in Science	Rockefeller University R3 - Enhancing Scientific Rigor, Reproducibility, and Reporting	University of California Los Angeles MOLBIO235 - Rigor and Reproducibility
National Institute of Health Enhancing Reproducibility through Rigor and Transparency	Stanford University Stanford Program on Research Rigor & Reproducibility	Columbia University Health Sciences Promoting Credibility, Reproducibility and Integrity in Research
National Science Foundation Companion Guidelines on Replication & Reproducibility in Education Research Framework to Improve Reproducibility, Replicability, and Robustness Social, Behavioral, and Economic Sciences Perspectives on Robust and Reliable Science	University of Florida R4I@UF - Rigorous Reproducible Responsible Research Integrity	Emory University Rigor and Reproducibility
	University of Minnesota The Many Faces of Reproducibility	University of California San Francisco Rigor and Reproducibility in Research
	Virginia Commonwealth University Data Science Lab	University of Florida Rigor and Reproducibility Seminar Series
		University of California San Diego Rigor and Reproducibility Workshop
		University of Washington Tools for Reproducible Research

## Discussion

Our study found that most CTSA hubs reported incorporating R & R content into their courses or had dedicated R & R training. This is likely a result of the NIH policies previously described. Incentives and recognition for these practices were reported as present in only five institutions. This was not surprising, as USA and international tenure and promotion criteria rarely specify R & R criteria or outcomes [13,14]. Our survey also revealed that respondents saw overlaps between R & R and topics embedded in either standard research methodology education or responsible conduct of research (RCR) training, and it was difficult to discern from survey results how respondents were making that distinction. We, therefore, believe the actual percentage of hubs with meaningful support for R & R is closer to the roughly 50%–70% formally using the terms “*rigor*” and “*reproducibility*” in courses or on their websites, rather than the 84% of PIs who stated that it was taught.

With this year being declared to be the “*Year of the Open Science*” in the USA [15] and the focus on development of open science practices and education, greater clarity will be needed regarding requirements for distinctive or integrated education or training in RCR, R & R, and open science [16,17]. Further efforts will be needed to facilitate accreditation of courses, and establishment of competencies for these specific terms. Greater transparency requires attention to data management processes before data are cleaned or analyzed. The importance of this has been demonstrated in a variety of many-lab and many-analyst projects in a wide range of applications, from cell-counting to imaging and psychology [18–20], as well as a variety of high-profile cases where conclusions were found to be unsupported only after close scrutiny of raw data [21–24]. It is also a focus of the 2023 NIH Data management requirements, which require a description of the pre-analytic data management process [8]. Openness and transparency are also necessary for proper assessment of rigor and for confirming reproducibility [25,26]. “*Research rigor*” requires attention not only to experimental design and conduct, including sample size implications, but to topics like hidden multiplicity, reporting of negative results, misinterpretations of p-values and statistical significance, and to the true strength of the evidence underlying research claims.

T32 requirements for R & R training, first instituted in May 2020, could have broad influence on R & R education at CTSA hubs as T32 grants are renewed. The effect on faculty practice is as yet uncertain, and these requirements do not extend to the array of research support services supported by CTSAs. Without broad-based integration at all levels of the research enterprise, the impact of trainee education could be limited. NCATS requirements and translational models should formally incorporate these principles, as there is substantial empirical evidence that it affects the translatability of both preclinical and clinical research.

Our study has a number of limitations. We did not receive responses from 11 of 61 (18%) CTSA hubs. As it is unlikely that non-respondents had more R & R activities than respondents, our reported rates are probably biased upwards. As we could only search publicly available websites, content on institutions' intranets was missed unless reported by survey respondents. Also, while respondents reported the existence of R & R-related training, we could not assess the coverage of R & R topics; we hope to collect such information in the future. One of the main motivations behind our study was to stimulate a broader discussion and establishment of standards that would make it clearer whether training satisfies RCR, GLP, or R & R requirements, and in which cases it could satisfy all three. We also did not ascertain the specifics of the monitoring and incentives that institutions reported. Furthermore, we did not assess the quality or extent of resources that the CTSAs provided.

We know of no other studies examining the support of rigor and reproducibility education and support provided by CTSA hubs. We hope this study facilitates sharing of R & R resources and best practices across the CTSA network and can serve as a baseline to monitor future progress. The collected resources reported herein are posted on the website of the Stanford Program for Research Rigor and Reproducibility (SPORR.stanford.edu) for use by the CTSA network and those outside. This web information will be updated with new information sent to SPORR [27].

## Supporting information

Axfors et al. supplementary material Axfors et al. supplementary material

## Supplementary material

The supplementary material for this article can be found at https://doi.org/10.1017/cts.2024.10.
