# Supplementary material for: Research rigor and reproducibility in research education: A CTSA institutional survey
Source: J Clin Transl Sci. 2024 Feb 1;8(1):e45. doi: 10.1017/cts.2024.10 (PMC10928701; doi:10.1017/cts.2024.10)
Supplement: Axfors et al. supplementary material [file S2059866124000104sup001.docx]

This is the supplementary file to the ***Research Rigor and Reproducibility in Research Education: A CTSA Institutional Survey*** by Cathrine Axfors, Mario Malički, Steven N Goodman

**Table of Contents:**

[Survey invite and response details	1](#_Toc144130109)

[Invitation and reminder emails	2](#_Toc144130110)

[Survey Text and Questions	4](#_Toc144130111)

[Detailed Results and Raw Data	7](#_Toc144130112)

[Website search and results	9](#_Toc144130113)

### Survey invite and response details

We conducted a survey to explore Rigor and Reproducibility (R&R) activities and resources of Clinical and Translational Science Awards (CTSA) Program funded US medical research institutions. To test the survey and allow its modification based on survey responses, we divided the 61 CTSA institutions into 3 groups, and sent the email invite to the first group on Jan 6, to the second on Jan 27, and to the third on Feb 16. Based on initial responses, we decided not to modify the initial set of questions. First reminder for groups 1 and 2 was sent on February 16, and for group 3 on Mar 1. Second reminder to all groups was sent on May 6, and third on 13 July. We then tried to reach the non-respondents by phone on August 28-29, and did a reminder call on Oct 10-11. Phone calls led to answers from additional 5 (29%) institutions. In the survey invite, reminders, and phone calls we advised the principal investigators of the CTSA grants to whom the survey was initially sent to forward the survey if they felt other individual(s) might be more knowledgeable of R&R activities at their institution. In total 29 (58%) responses were from individuals who were forwarded the invite. While initially we did not fill out the survey for our own institutions, on 6 December we added the answers for Stanford to be available in the (raw) and the shared dataset. Answers were filled out by Mario Malički. Response rate (without Stanford) was 82% (49 of 60 institutions). For 2 Institutions we had responses from 2 individuals, and we merged those. In case individuals provided different answers, we used the more “beneficial” answer, e.g., if one respondent said yes that monitoring was implemented and the other no, we used the yes answer as the final one.

### Invitation and reminder emails

**Invitation email:**

Subject: Survey on Research Rigor and Reproducibility Programs at CTSA-funded Institutions

Dear Dr. <Last Name>,

We are writing from the Stanford Center for Clinical and Translational Research (Spectrum), funded through the Stanford CTSA, about a CTSA-sponsored project to help us understand what activities CTSA-funded institutions are implementing to improve research rigor and reproducibility (R&R*) at their sites. As you know, the NIH and other major funding agencies and journals are increasingly calling for formal training and support for practices that enhance R&R. These institutional activities can include website links to resources, education, training, monitoring, support and other measures. Pilot work indicates that such efforts either haven’t begun or are in early stages at most institutions.

Through this initiative, we aim to create a forum where R&R activities and resources can be shared between CTSA institutions. We will present the results of this survey in a report for the CTSA network and published in a peer-reviewed journal, without identifying individual institutions.

This invitation is being sent to all CTSA Principal Investigators. We would greatly appreciate your completing our 5-10 minute survey on this topic, or passing it on to someone at your institution who is most knowledgeable about this. (That mechanism is on the survey.) We are interested in any such institutional activities, regardless of whether they are CTSA funded or not. Click to access the survey

Thank you very much for your time. Please reach out to us if you have any questions. We will send a reminder in 10-14 days if we haven’t heard from you. Thanks in advance for your help.

Sincerely,

Steve Goodman

Steven Goodman, MD, MHS, PhD

Associate Dean and Professor of Epidemiology and of Medicine

Director, Stanford Program on Research Rigor and Reproducibility (SPORR)

Director, Stanford Translational Workforce Development program

Co-Director, Stanford KL2 Program

Stanford University School of Medicine

<http://sporr.stanford.edu/>

Cathrine Axfors, MD, PhD

Postdoctoral Fellow, Meta-Research Innovation Center at Stanford (METRICS)

[caxfors@stanford.edu](mailto:caxfors@stanford.edu)

* Rigor and reproducibility (R&R), working definition

Research rigor and reproducibility are not completely separable, but in general “rigor” refers to scientific practices that can improve the reliability of results, and is mainly focused on proper design, conduct and analysis, including validation of the laboratory materials and instruments. “Reproducibility” captures the ability to understand the scientific procedures used in a study through proper and complete reporting, data management and documentation.

**Reminder email:**

Subject: Reminder about rigor and reproducibility survey

Dear Dr. <Last Name>

I hope this email finds you well. This is a friendly reminder about a request previously sent to you, as PI of the <INSTITUTION>, to fill out a brief survey about any activities at <INSTITUTION> designed to improve research rigor and reproducibility (R&R), or to redirect the survey to someone else at your institution who would know the most about this. (That mechanism is on the survey.) If we missed your response, apologies and thank you!

Getting a national picture of R&R activities, whether CTSA funded or not, is of great interest to NCATS and to the NIH. We plan to use these findings to inform institutions of what others are doing, to help them create or strengthen their own programs. We will share the results, anonymized to institution, with all respondents and with NCATS. We expect that many or most institutions will currently have only nascent efforts or may not have yet started down this road.

The original invitation is reprinted below, and the survey can be found here: **Click to access the survey**

Please don’t hesitate to reach out if you have any questions. We are very thankful for your time.

Sincerely,

Steve

Steven Goodman, MD, MHS, PhD

Associate Dean and Professor of Epidemiology and of Medicine

Director, Stanford Program on Research Rigor and Reproducibility (SPORR)

Director, Stanford CTSA Translational Workforce Development program

Co-Director, Stanford CTSA KL2 Program

Stanford University School of Medicine

**----**

Original invite was reprinted below.

**Final reminder email:**

Subject: Final reminder about CTSA rigor and reproducibility survey

Dear Dr. <Last Name>,

This is a final reminder about a request previously sent to you to fill out a short survey of CTSA institutions about their activities related to research rigor and reproducibility. [CLIC](https://mailchi.mp/c93c65fa473c/clic-news-roundup-june-14-5673657?e=81e56cc640#CTSA%20News) recently linked to an [article](https://med.stanford.edu/spectrum/about-spectrum/news/stanford-launches-ctsa-wide-survey-on-rigor-and-reproducibility) about the study. We would greatly appreciate if you could fill out the survey or pass it on to anyone at your institution who is knowledgeable about rigor and reproducibility activities, even if not directly supported by your CTSA grant. We are equally interested in the presence or absence of such programs, so please respond even if your institution has not set anything up yet. Otherwise, the responses could be highly skewed.

The original invitation is reprinted below, and the 5-10 minute survey can be found here: Click to access the survey

Please don’t hesitate to reach out if you have any questions. We are very thankful for your time. If you have already responded, thanks!

Sincerely,

Steve

Steven Goodman, MD, MHS, PhD

Associate Dean of Clinical and Translational Research,

Professor of Epidemiology and of Medicine

Director, Stanford CTSA Translational Workforce Development program

Co-Director, Stanford CTSA KL2 Program

Director, Stanford Program on Research Rigor and Reproducibility ([SPORR](http://sporr.stanford.edu/))

Stanford University School of Medicine

**----**

Original invite was reprinted below.

### Survey Text and Questions

Research Rigor and Reproducibility Survey

Survey on Research Rigor and Reproducibility Programs at CTSA-funded Institutions

A 5-10 minute survey

This survey is being administered by the Stanford Center for Clinical and Translational Research (Spectrum), a CTSA Program Hub. The purpose of the survey is to understand what kind of activities host institutions for CTSA Program Hubs might be implementing to improve practices related to research rigor and reproducibility (R&R) at their sites.

We are interested in such activities even if not directly supported by the CTSA. These institutional activities can include education, training, monitoring, support, accountability measures or other measures. Pilot work indicates that such activities either haven’t begun or are in early stages at most institutions.

Results will be reported back to all institutions who respond. There will be no identification of individual institutions in any reports from this survey unless specific permission is later granted. The Institutional Review Board at Stanford University has confirmed that this project does not meet the definition of human subjects research. This survey should take only 5-10 minutes to complete. Thank you in advance for your help.

If there is someone else we should be contacting to get this information, we have included a field where you can provide their contact information.

If you have any questions or comments about this survey, please contact Dr. Cathrine Axfors (caxfors@stanford.edu) or Dr. Steven Goodman (steve.goodman@stanford.edu).

What is research rigor and reproducibility (R&R)?

Research rigor and reproducibility are not completely separable, but in general “rigor” refers to scientific practices that can improve the reliability of results, and is focused on proper design, conduct and analysis, including validation of experimental materials and instruments.

“Reproducibility” captures the ability to understand the scientific procedures used in studies through complete reporting, good data management and thorough documentation.

Efforts to improve research R&R extend beyond those traditionally used to prevent research misconduct, i.e. falsification, fabrication and plagiarism, which are under the purview of institutional Research Integrity Officers.

1. Institution and contact information

Institution name

First name

Last name

Preferred email address

Please confirm your preferred email address

Title/role at your institution

Title/role at your CTSA (if applicable)

2. Additional respondent

Would you like to suggest an additional respondent at your institution to fill out this survey?

First name, additional respondent

Last name, additional respondent

Email address, additional respondent

Title/role, additional respondent

Reason for suggesting this respondent

3. Institutional activities to improve research rigor and reproducibility (R&R)

We would like to capture information about activities at your institution regardless of whether they are CTSA-funded or not. All questions below refer to activities at the school, institution, or university levels, rather than activities restricted to individual departments or divisions.

3.1) Does your institution have a website or webpage with links to online R&R resources or related government requirements?

3.1a) If possible, could you please provide the URL for that webpage below?

3.2) Has your institution incorporated R&R training into existing courses or seminar series?

3.2a) If there are any related websites describing these courses, please provide URLs here:

3.2b) Approximately how many hours of R&R training?

3.3) Does your institution have any training or educational offerings exclusively devoted to research R&R?

3.3a) If there are any related websites describing these courses, please provide URLs here:

3.3b) Approximately how many hours of R&R training?

3.4) Does your institution have a monitoring program to assess the implementation of R&R practices (e.g. related to design, protocol development and sharing, data management, results reporting, data archiving or sharing, or open science)?

3.5) Does your institution have a program that provides technical assistance or other support for implementation of R&R practices?

3.6) Does your institution have a program that recognizes and/or incentivizes use of best R&R practices?

3.7) Please share any other efforts in supporting R&R practices at your institution that are not captured above, such as activities organized by specific departments or divisions:

3.8) Is participation in any of the existing activities mandatory for any members of your institution?

3.9) What are the main funding sources of the activities described above?

3.10) Documents

We are interested in the organization and activities of any program to improve R&R at your institution. If possible, please share any relevant documents that describe the content or organization of your program or initiative. Before sending in the form, please wait for the files to upload.

Drag and drop files here or browse files

3.11) If there are any websites related to or describing the program, please provide URLs here:

3.12) Are you part of the team that organizes the program?

If you have additional comments about the activities to improve research R&R at your institution, please enter them here:

4. Updates about this project

Are you interested in joining our mailing list?

Submit Survey (with option to send responses to the respondee).

Upon hitting the submit button the following message is displayed:

Your response has been captured. Thank you for helping us understand and accurately represent the activities at your institution.

### Detailed Results and Raw Data

As the raw data includes names, emails, and open-ended comments that share confidential information, original raw data is available only from the authors on the request. The cleaned dataset can be found at [https://doi.org/10.25740/rh435ns0610.](https://doi.org/10.25740/rh435ns0610.%20) Note: 6 institutions uploaded documents as part of question 3.10. The information provided in those documents was incorporated in the results below, the documents are only available upon request as part of the raw data.

Responses are presented as the number and percentage of responding institutions (including Stanford). All data was explored using MedCalc v.20.123 (RRID:SCR_015044), and is reported in Supplementary Table 1 below.

Supplementary Table 1.

| **Question** | **Response** | **n (%)** |
| --- | --- | --- |
| Does your institution have a website or webpage with links to online R&R resources or related government requirements? | Yes | 28 (56) |
|  | listed website | 24 (48) |
|  | No or not that I know of | 16 (32) |
|  | Limited or in development | 6 (12) |
|  | listed website | 2 (4) |
| Has your institution incorporated R&R training into existing courses or seminar series? | Yes | 42 (84) |
|  | listed website with course(s) or program description* | 12 (24) |
|  | No or not that I know of | 4 (8) |
|  | Limited or in development | 4 (8) |
|  | listed website with course(s) or program description* | 1 (2) |
| Hours of training (Md, IQR, Note: when more courses were listed, average was taken, N=21) | 3 (2 to 8) | |
| Does your institution have any training or educational offerings exclusively devoted to research R&R? | Yes | 34 (68) |
|  | listed website with course(s) or program description | 13 (26) |
|  | No or Not that I know of | 16 (32) |
|  | Limited or in development | 3 (6) |
| Hours of training (Md, IQR, Note: when more courses were listed, average was taken, n=18) | | 7 (2 to 12) |
| Does your institution have a monitoring program to assess the implementation of R&R practices? | Yes | 15 (30) |
|  | In development | 4 (8) |
|  | No or not that I know of | 31 (62) |
| Does your institution have a program that provides technical assistance or other support for implementation of R&R practices? | Yes | 27 (54) |
|  | In development | 7 (14) |
|  | No or not that I know of | 16 (32) |
| Does your institution have a program that recognizes and/or incentivizes use of best R&R practices? | Yes | 5 (10) |
|  | In development | 6 (12) |
|  | No or not that I know of | 39 (78) |
| Is participation in any of the existing activities mandatory for any members of your institution? (N=42) | Yes | 28 (67) |
|  | No, all activities are elective | 11 (26) |
|  | Not that I know of | 3 (7) |
| What are the main funding sources of the activities described above? (Note: Total exceeds 100 as some institutions listed more than one source of funding. N=38) | NIH (e.g. CTSA, T32) | 20 (53) |
|  | Institution | 32 (84) |
|  | Other (services or tuition) | 3 (8) |
|  | Don’t know or NA | 3 (8) |
|  | No funding | 1 (3) |
| Website(s) with related to or describing the program on R&R. | | 13 (26) |
| Are you part of the team that organizes the program? (N=34) | Yes | 20 (58) |
|  | No | 14 (42) |

### Website search and results

We expanded the raw data by conducting a literature search of institutions' websites. Websites were first searched using their internal search engines with the term *rigor and reproducibility,* Additionally, we also used Google with 3 search strategies (search?q=site:UNI “*rigor and reproducibility”,* search?q=site:UNI “rigor & reproducibility”, and search?q=site:UNI “r&r”). (Note: UNI was substituted with university website, and ampersand, i.e. &, with “%26” in order to avoid it being interpreted as search syntax character). First 4 webpages of results were checked (if available). All websites searches were conducted from 15 to 25 August 2022, except for search of University of Texas Hlth Science Center at San Antonio which was re-done on Nov 15 as during data cleaning we discovered that we originally mistakenly searched Uni. of Texas Houston website twice instead. Data from the website search is incorporated in our shared data file.

Results based on the survey and supplemented by the data search are shown below. Data is reported as number and percentage of 61 CTSA institutions. All data was explored using MedCalc v.20.123 (RRID:SCR_015044), and is reported in Supplementary Table 2 below.

We recorded some of the answers respondents provided and supplemented them using the website searches, based on the following rules:

1. Resource page has to be or have a dedicated (sub)section for **external** rigor or reproducibility resources.
2. For incorporated training institution has to have a **publicly available website** with a course whose descriptions includes addressing **rigor or reproducibility**.

Supplementary Table 2.

| **Resources** | **Classification** | | **n (%)** |
| --- | --- | --- | --- |
| Webpage with links to external R&R resources (all links available at [SDR](https://purl.stanford.edu/rh435ns0610)) | Yes | | 34 (56) |
|  | University-wide (e.g., Library website) | | 22 (36) |
|  | Department-wide (e.g., Dept of Surgery) | | 4 (7) |
|  | Grant training focused (e.g., Office of Grant Support) | | 8 (13) |
|  | No | | 27 (44%) |
| Institutions with a website with a publicly visible incorporated R&R training | | | 33 (54%) |
| Devoted Training | | Yes | 34 (56) |
|  |  | Course materials/videos  available | 8 (13) |
|  |  | No | 27 (44) |
| Program with description indicating focus on rigor or reproducibility | | | 7 (11) |

The identified support services are presented in Supplementary Table 3 below

Supplementary Table 3. Description and links of support services.

| **Support Type** | **Description** | **Link** |
| --- | --- | --- |
| Computational Reproducibility | Results Reproduction (R-Squared) is a service that computationally reproduces the results of your research to ensure Reproducibility and Transparency , Grant review services for RR | [https://socialsciences.cornell.edu/research-support/R-squared, https://library.weill.cornell.edu/research-support/grant-editing](https://socialsciences.cornell.edu/research-support/R-squared) |
| Consulting | Reproducibility and rigor checks | [https://www.uab.edu/norc/cores/design-analytics-core, https://web.archive.org/web/20210120160721/https://www.uab.edu/norc/cores/design-analytics-core](https://www.uab.edu/norc/cores/design-analytics-core) |
| Consulting | VCU Libraries data management group provides resources on its site, engages in consultation and hosts a webinar series. | per survey response |
| Consulting | "Reproducibility Librarian" available in Health Science Center Library, available for free consultations | [https://news.cci.fsu.edu/jobs-and-internships/cci-job-board/mls-jobs/reproducibility-librarian-uf-libraries/ (link to old add)](https://news.cci.fsu.edu/jobs-and-internships/cci-job-board/mls-jobs/reproducibility-librarian-uf-libraries/%20(link%20to%20old%20add)) |
| Consulting | This ensures that our Center resources have the greatest opportunity to support high-impact studies in aging research and also meets an important goal of enhancing rigor and reproducibility in biomedical research. | <https://halo.dlmp.uw.edu/uwhalo/nsc/nathan-shock-center-cores/artificial-intelligence-and-bioinformatics/> |
| Consulting | Biostatistics consulting center evaluates R&R plan (for money) | <https://www.sph.emory.edu/departments/bios/documents/BCC%20Rate%20Sheet%20as%20of%20Sept2016.pdf>, <https://www.cores.emory.edu/bcc/_includes/documents/sections/services/bcc-tier-and-hourly-rates-sept2020.pdf> |
| Consulting + Lectures | Library - work with individuals, research groups, and departments to advise on many aspects of reproducibility and rigor. If you have any questions reach out to the subject librarian for your area. | <https://www.lib.umn.edu/services/reproducibility> |
| Lectures | Staff provide free lectures and presentations for graduate students, university departments, and research project teams on data management best practices, standards and tools. We also offer sessions on reproducible research practices to support open and transparent science. | <https://odum.unc.edu/archive/> |
| Training and mentoring | Aim 2: Outreach to educate SCC researchers on the fundamentals of biostatistics, data management, and research rigor and reproducibility by enforcing statistical operating standards in research protocol reviews at SCC. BSR faculty are actively involved in training and mentoring students, residents, fellows, and junior faculty in statistical concepts through roles as course-masters or co-instructors in programs awarding formal degrees (e.g., MS, PhD, MD). BSR faculty apply statistical and rigor/reproducibility standards to SCC research to protect the scientific integrity of research and patient safety. This is accomplished at a study-specific level through their role on independent Data Safety and Monitoring Committees for SCC investigator-initiated, multi-site clinical trials. At an institutional level, BSR faculty apply these standards as reviewers on SCC’s Protocol Review and Monitoring Committee (PRMC), Siteman Investment Program (SIP) scientific study section, and the SCC Quality Assurance and Safety Monitoring Committee (QASMC). Key components of BSR’s protocol review include evaluation of study design, statistical power/sample size calculations, and futility/efficacy/toxicity monitoring rules. Statistical analysts follow standard operating procedures to ensure reproducibility. | <https://siteman.wustl.edu/research/shared-resources-cores/biostats-core/> |
